# Supplementary material for: The association between alcohol consumption and herpes simplex virus type 2: A cross-sectional study from national health and nutrition examination survey 2009–2016
Source: PLoS One. 2024 Jul 24;19(7):e0307702. doi: 10.1371/journal.pone.0307702 (PMC11268616; doi:10.1371/journal.pone.0307702)
Supplement: S1 File — (DOCX) [file pone.0307702.s001.docx]

Table S1. Association of covariates and HSV-2.

| **Variables** | **OR (95% CI)** | ***p*** | **Variables** | **OR (95% CI)** | ***p*** |
| --- | --- | --- | --- | --- | --- |
| **Age(years)** | 1.06 (1.06~1.07) | <0.001 | **Smoking status, n (%)** |  |  |
| **Age(years), n (%)** |  |  | Never | Ref |  |
| 20-24 | Ref |  | Former | 1.25 (1.06~1.48) | 0.008 |
| 25-30 | 1.77 (1.36~2.3) | <0.001 | Current | 1.91 (1.68~2.18) | <0.001 |
| 31-40 | 3.12 (2.47~3.94) | <0.001 | **Frequency of Condom Use, n (%)** |  |  |
| 41-49 | 4.9 (3.88~6.18) | <0.001 | Never | Ref |  |
| **Gender, n (%)** |  |  | Less than half of time | 0.85 (0.7~1.02) | 0.086 |
| Female | Ref |  | About half of time | 1.05 (0.83~1.34) | 0.673 |
| Male | 0.48 (0.43~0.54) | <0.001 | More than half of time | 0.79 (0.63~0.99) | 0.043 |
| **Race/Ethnicity, n (%)** |  |  | Always | 0.87 (0.75~1) | 0.047 |
| Mexican American | Ref |  | **Diabetes, n (%)** |  |  |
| Non-Hispanic black | 5.16 (4.21~6.31) | <0.001 | No | Ref |  |
| Non-Hispanic white | 1.12 (0.92~1.37) | 0.275 | Yes | 2.14 (1.7~2.69) | <0.001 |
| Others | 1.22 (0.98~1.52) | 0.07 | **Hypertension, n (%)** |  |  |
| **Marital status, n (%)** |  |  | No | Ref |  |
| Living alone | Ref |  | Yes | 1.81 (1.59~2.06) | <0.001 |
| Married or living with a partner | 0.61 (0.54~0.68) | <0.001 | **CKD, n (%)** |  |  |
| **Education level, n (%)** |  |  | No | Ref |  |
| Less than high school | Ref |  | Yes | 1.55 (1.26~1.91) | <0.001 |
| High school or GED | 1 (0.85~1.19) | 0.955 | **COPD, n (%)** |  |  |
| Above high school | 0.61 (0.52~0.71) | <0.001 | No | Ref |  |
| **PIR, n (%)** |  |  | Yes | 2.45 (1.68~3.57) | <0.001 |
| ≤1.3 | Ref |  | **CVD, n (%)** |  |  |
| 1.3-3.5 | 0.8 (0.7~0.91) | 0.001 | No | Ref |  |
| ＞3.5 | 0.52 (0.45~0.61) | <0.001 | Yes | 2.56 (1.89~3.47) | <0.001 |
| **BMI(Kg/m2)** |  |  | **Canacer, n (%)** |  |  |
| ＜25 | Ref |  | No | Ref |  |
| ≥25 | 1.7 (1.49~1.94) | <0.001 | Yes | 1.65 (1.21~2.27) | 0.002 |
| **Health Insurance Coverage, n (%)** |  |  |  |  |  |
| No | Ref |  |  |  |  |
| Yes | 0.8 (0.71~0.91) | <0.001 |  |  |  |

Abbreviations: OR odds ratio; CI confidence interval.

Figure S1 The relationship between alcohol consumption and HSV-2 in various subgroups
